# Are organic acids really related to the sour taste difference between Chinese black tea and green tea?

**DOI:** 10.1002/fsn3.2823

**Published:** 2022-04-19

**Authors:** Xiang Zhang, Xiao Du, Ying‐zheng Li, Cong‐ning Nie, Cong‐ming Wang, Jin‐lin Bian, Fan Luo

**Affiliations:** ^1^ Sichuan Academy of Agricultural Sciences of Tea Research Institute Chengdu China; ^2^ 12529 Sichuan Agricultural University Chengdu China

**Keywords:** sensory, sour, taste, tea

## Abstract

*Sour* is an important taste in some foods, beers, and teas; organic acids, in particular, are thought to play a key role in the formation of the sour taste of beer. It has been generally thought that organic acids also contribute to some teas tasting sour. In this study, through sensory evaluation experiments with black tea (BT) and green tea (GT), the difference in the sour taste of BT and GT was quantitatively characterized. Then the organic acids in the two types of tea were identified and quantified via high‐performance liquid chromatography (HPLC) with taste activity value (TAV) analysis. The results showed that both teas had 12 identical common organic acids (including 11 taste‐active components), but the results of the TAV analysis were not consistent with those of the sensory evaluation. Therefore, there is no direct relationship between organic acids and the acidity in BT and GT. It is related to the interaction between organic acids and other substances, pH value, or other sour substances in tea infusions. The mechanism of the disappearance of sourness in tea infusions was also discussed. These results help us to understand the correlation between tastes in teas.

## Graphical abstract

Organic acid is one of the important taste substances in tea infusions. In this study, the acidity and organic acid content of black tea (BT) and green tea (GT) were studied and it was found that the acidity in BT and GT was not directly related to organic acids, but was related to the interaction between other sour substances. The mechanism of the disappearance of sour in tea was discussed, which may be due to the masking or retronasal sniffing.

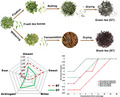

## 1 INTRODUCTION

Tea (*Camellia sinensis*) is one of the top three most popular nonalcoholic beverages in the world (Yue et al., 2017). According to its sensory characteristics and production process, tea can be classified as black, green, yellow, white, dark, and oolong (Nie et al., 2019). Aroma and taste are the main factors in high‐quality tea (Dong et al., 2017), and the dynamic differences in their content, proportion, and composition provide the focus of the current study.

Different types of tea have different taste characteristics (Scharbert & Hofmann, 2005). Four of the five basic taste types, sour, sweet, bitter, and umami (Li & Liu, 2015), can be identified independently of tea. (It is generally accepted that, in most cases, a salty taste cannot be identified in tea alone.) In the above four taste types, a moderate acidity, which is mainly produced during fermentation (Shi, 2010), is thought to be common in black tea (BT), but high acidity concentrations will reduce the harmony of the tea taste (Li & Liu, 2015) and could be considered a taste deficiency, especially in green tea (GT).

The taste of tea mainly comes from nonvolatile substances; therefore, the study of nonvolatile components in tea is undoubtedly important. These elements affect the quality of the characteristics and nutritive value of tea infusions. A previous study showed that the concentration of all organic acid species played a key role in the intensity of acidified foods. Thus, organic acids are always viewed as important factors in the sour taste of tea infusions. However, these literature do not contain data about the organic acid components and sour taste characteristics of tea, even though more than 100 nonvolatile components (including catechins, free amino acids, soluble sugars, flavonoids, flavonoid glycosides, and organic acids) are present in tea (Dandan, 2016). For the past 3 decades, research on the production and manufacturing processes of tea has suggested that BT commonly has a moderate sour taste (Lin et al., 2017), while GT does not. Thus, it is highly important to study the sensory differences and materials in the taste components of both BT and GT, not only because similar nonvolatile components play a different role in sensory evaluation but also because the differences in these taste components can reveal the taste–taste interactions of sensory evaluation.

The objective of this study was to explore whether organic acids play a crucial role in the sour taste of tea. To address this question, a taste profile of BT and GT (free of pesticides and other pollutants) was created. Then the concentration of organic acid species in BT and GT was detected via high‐performance liquid chromatography (HPLC). The results uncovered the true relationship between sour taste and organic acid in tea infusions while providing new insight into the sour taste of BT and GT.

## 2 MATERIALS AND METHODS

### 2.1 Chemicals

The following materials were purchased from Dikma Technologies: oxalic acid, pyruvic acid, dl‐tartaric acid, malonic acid, malic acid, lactic acid, fumaric acid, maleic acid, acetic acid, citric acid, succinic acid, and gluconic acid (≥99.0%). Pure water, with a pH of 6.67 (±0.05), was used in all experiments.

### 2.2 Tea samples

#### 2.2.1 Manufacturing processes of BT and GT

**FIGURE 1:**
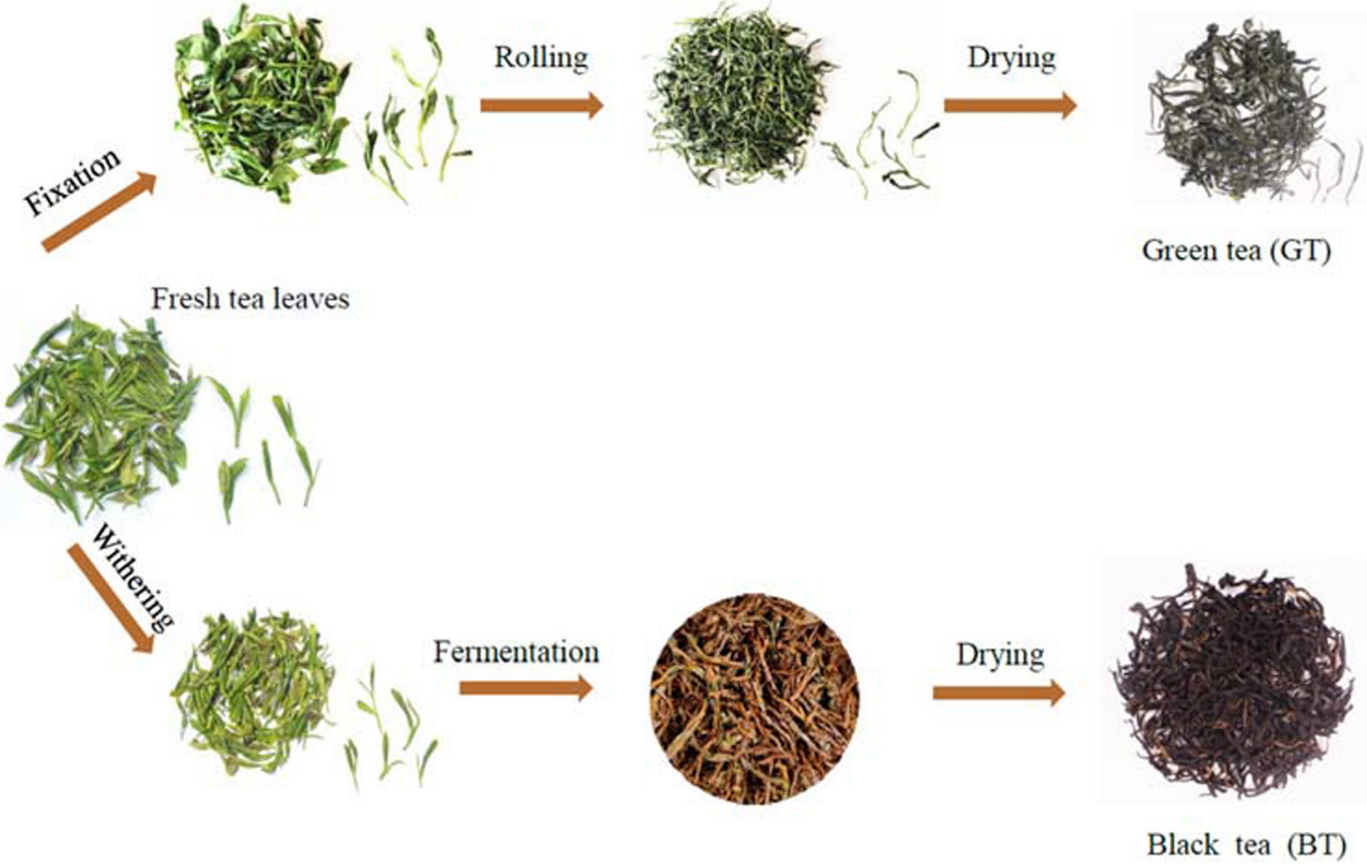
Manufacturing processes of black tea (BT) and green tea (GT)

Fresh leaves were plucked from a tea garden in Gaoxian, Yibin City, Sichuan Province (N28°47′, E104°36′) in May 2019. The picking standard was one bud and one to two leaves; the tea cultivar was a mesolobular population from Sichuan with *Camellia sinensis var. sinensis* leaves. All the fresh leaves were picked on the same day, and then the tea samples were immediately produced by the Chuan Hong Group Co. Ltd. in Yibin. As evident from Figure 1, BT and GT were processed.

The GT manufacturing process was as follows:

Spread: Freshly plucked leaves were exposed to shade for about 2–4 hr to ensure a reduction in moisture of about 20%.

Fixation: Also known as inactivation, fixation is the process of deactivating enzymes with high temperatures (200℃ for setting and 90 ± 2℃ for the actual temperature of the leaves) and brief durations (70–90 s), using a rotary hot‐blast air roller fixation machine.

Rolling: A technique that uses a tea twisting machine (55 ± 5 r min^−1^ rotation rate setting at a duration of 30 ± 5 min) to break leaf cells, rolling also can decide the fine and tight degree of tea leaf strip rope.

Drying: A roller stir‐fry machine was used to dry and shape the tea, ensuring it was curly, until the moisture content was <7%.

The BT manufacturing process was as follows:

Solar wilting (SW): Freshly plucked leaves were exposed to sunlight for about 4–6 hr to make sure the water content went down to 50% (±5%).

Rolling and fermentation: A tea twisting machine (55 ± 5 r·min^−1^ rotation rate setting at a duration of 30 ± 5 min) was used to break the leaf cells and then the leaves were covered with a wet gauze for natural fermentation.

Drying: A roller stir‐fry machine was used to dry and shape the tea, ensuring it was curly until the moisture content was <7%.

After manufacture, the tea samples were stored in a refrigerator at −40℃.

#### 2.2.2 Preparation of the tea infusion

Three grams of each tea sample was infused in 150 ml of boiling water and maintained for 5 min for evaluation. It should be noted that the best tea infusion temperature for sensory evaluation is 45–65℃.

### 2.3 Quantitative sensory evaluation of tea infusions

The samples of BT and GT were submitted to a sensory evaluation called quantitative descriptive analysis by a panel of seven professional assessors (four female and three male; Chen et al., 2009; Qi et al., 2018). All the evaluators had prior training in recognizing, describing, and quantifying the intensities of different taste characteristics with standard reference liquids and tea infusions (Cadwallader and Song, 2008; Huanlu & Xia, 2008). Using a system of intensity scoring (see Table 1), seven evaluators were asked to rank the tastes of umami, sweet, bitter, sour, or astringent infusions on a scale of 0 to 3, where 0 = absent, 0.5 = slight, 1 = weak, 1.5 = noticeable, 2 = obvious, 2.5 = strong, and 3 = extreme. The arithmetical mean values of the tasting panel scores represent the range of the sour taste.

**TABLE 1:** Definition and numerical scale (0–3) of taste sensory factors in Chinese tea

Factors	Define	Reference	Numerical scale
Umami	Fresh and refreshing taste	l‐(+) Sodium glutamate	Extreme, 3 Strong, 2.5 Obvious, 2 Noticeable, 1.5 Weak, 1 Slight,0.5 Absent, 0
Sweet	A sweet taste	Alpha‐d‐Lactose monohydrate	
Bitter	A bitter taste and aftertaste	Saponin	
Sour	One of the basic tastes, produced after fermentation	Citric acid	
Astringent	A thick or scraping feeling on the tongue after consumption, like eating raw persimmon	Epigallocatechin gallate	

Note

a. The selection of sensory factors was based on the literature (Chen & Zhang, 2007; Toelstede & Hofmann, 2008), and the terms were defined by an experienced professor. b. The evaluation was conducted on a 7‐point scale; the number range from extreme to absent is 3~0, with each 0.5 divided into a gradient. c. Sensory evaluation was repeated three times for each evaluator.

### 2.4 Extraction and quantitation of organic acids in tea

#### 2.4.1 Sample preparation

A standard solution of 100 mg·ml^−1^ organic acid was pipetted into a conical flask, then it was diluted at different volumes with high‐purity water (Milli‐Q) to obtain standard solutions of different concentrations at 0.01, 0.1, 1, 10, and 50 mg·ml^−1^.

Three grams of tea was brewed with 150 ml of boiling water for 5 min, filtered with 0.22‐µm membrane, and concentrated to 30 ml, and injected into the machine.

#### 2.4.2 Analysis of contents of organic acids in tea samples

Instrument: Agilent 1260 high‐performance liquid chromatograph (HPLC) with diode array detector. Chromatographic conditions: Spursil C18 column (250 × 4.6 mm, 5 μm). Flow rate: 0.8 ml·min^−1^; sample volume: 10 μl. Mobile phase: 25 mM potassium dihydrogen phosphate, adjusted to pH of 2.5. Detection wavelength, 210 nm.

### 2.5 Determination of pH in tea infusions

According to the sensory evaluation method, BT and GT were brewed, respectively, to obtain the tea infusions. When the temperature of the infusions fell to room temperature (25℃), the pH levels were measured with a pH meter.

### 2.6 Statistical analysis

#### 2.6.1 Analysis of contents of organic acids in tea samples

##### Degree of precision

Standard samples with a mixed standard concentration of 10 mg·ml^−1^ were injected into the HPLC‐PDA (phosphate‐array detector) six consecutive times; the relative standard deviation (RSD) of each component in the standard solution was between 0.23% and 0.91%, confirming the instrument's precision.

##### Repeatability

Six samples were repeatedly processed in HPLC‐PDA, and the RSD of each component was calculated between 1.21% and 2.51%, indicating consistency of method.

##### Computational formula

The mixed standard solution was diluted in a series of standard solutions, step‐by‐step, as described above. The concentration *x* (mg·ml^−1^) of the standard solution (*x*‐coordinate) and the peak area ratio *y* of the reference product (*y*‐coordinate) were used to draw the standard curve and calculate the content of the target substance in the sample. All data were processed through an Agilent chemical workstation (https://www.nist.gov/) and Excel 2010.

#### 2.6.2 Calculation method of TAV

Our research included taste thresholds from various pieces of literature and monographs, as well as taste description data from published articles and the Chemical Book (https://www.chemicalbook.com/). The contents of the taste‐active compounds were analyzed using HPLC. Furthermore, TAV, or the ratio of the concentration of substances to the taste threshold, was calculated using the formula TAV = *C*/*T*, where *C* represents the concentration of taste components and *T* represents the taste thresholds of the corresponding components.

Taste studies in general have shown that if the TAV were equal to or greater than 1, this would usually mean the individual taste components had a certain impact on the overall taste, when in reality they had little or no impact. The higher the TAV, the greater the individual taste components contributed to overall taste.

Excel 2010 and Plot.ly (https://chart‐studio.plot.ly/feed) were used to analyze the data and draw diagrams.

## 3 RESULTS AND DISCUSSION

### 3.1 Difference in the intensity of sour taste between GT and BT

**FIGURE 2:**
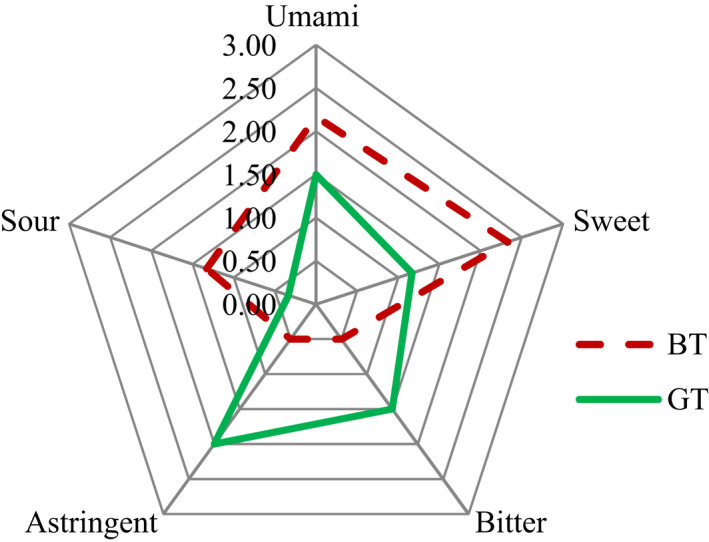
Taste profile for sensory tea samples. Black tea's (BT’s) scores for umami, sweet, bitter, sour, and astringent were 2.17, 2.33, 0.50, 0.50, and 1.33, respectively; green tea's (GT’s) scores were 1.50, 1.17, 1.50, 2.00, and 0.33, respectively

Sensory characterization of quantitative sensory evaluation is a useful way to describe the comprehensive details of many food characteristics, such as the taste qualities of chocolate and coffee. The taste qualities of BT and GT were described in this experiment (see Table 1), and the umami, sweet, bitter, sour, and astringent factors were quantified; these results were then used to obtain the taste profile shown in Figure 2, which intuitively showed the differences between taste characteristics of BT and GT.

As shown in Figure 2, the overall taste intensities were divided into five basic taste characteristics (see Table 1). This process revealed that BT, with its characteristic sweetness, had higher intensities of overall taste than GT, which has basic astringent and umami qualities according to sensory reference standards.

The most obvious flavor characteristics of BT are strong sweetness (sensory score = 2.33), obvious umami (2.17), a noticeable sour taste (1.33), slight bitterness (0.50), and an astringent taste (0.50). In terms of GT, the most obvious flavor characteristics are astringent (2.00) and noticeable umami and bitterness (each 1.50). The sweetness of GT was weak (1.17), and a sour taste was almost nonexistent (0.33).

Quantitative sensory evaluation showed that BT and GT have very different acidity levels. As demonstrated by Wan (2005), the sweetness of a tea infusion must be in direct proportion to the soluble sugar content, and the acidity should be in direct proportion to the organic acid content. For this reason, the effects of acid components (mainly organic acids) on the overall taste of BT and GT were studied in the two samples.

### 3.2 Composition of organic acid components in BT and GT

**FIGURE 3:**
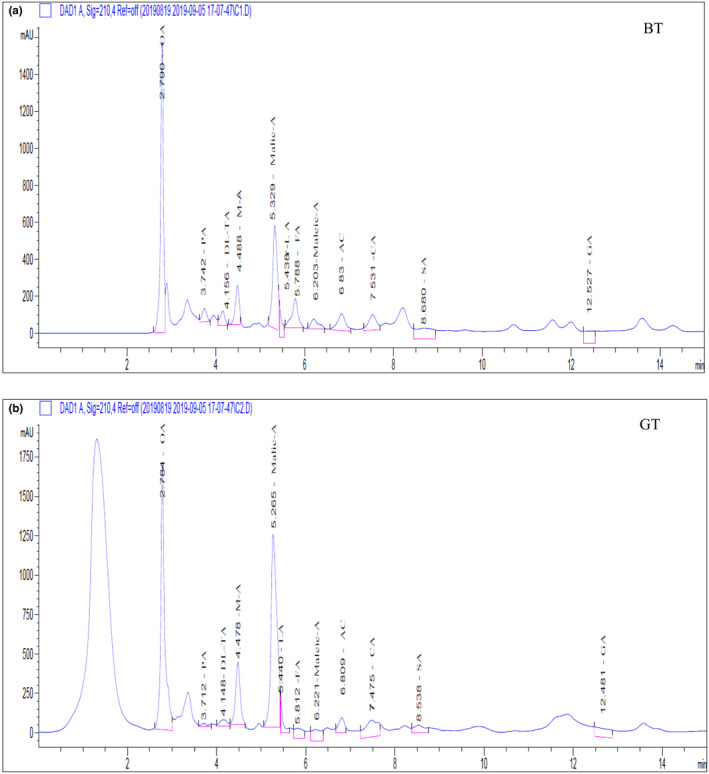
Total ion chromate grams of organic acid compounds in black tea (BT) and green tea (GT). (a) The *X*‐axis represents the retention time (min), and the *Y*‐axis represents the Response value (mAU). (b) The citric acid were marked with English acronyms

**FIGURE 4:**
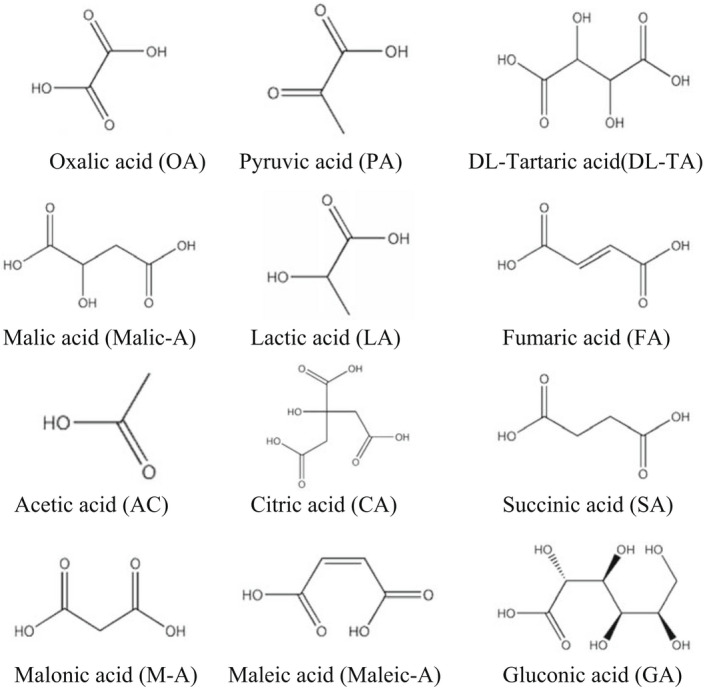
The structures of common organic acids

The total ion chromate grams of organic acids of the two samples appear in Figure 3. The separated and identified organic acid components and the taste description of the two samples appear in Table 2. Finally, the organic acid components were also analyzed for TAV; these detailed data appear in Table 2 and Figure 4.

**TABLE 2:** Sour taste (organic acids) components’ data from black tea (BT) and green tea (GT) by taste activity value (TAV) calculation

No.	Organic acids components	Content: mg·g^−1^		Taste threshold: mg·g^−1^	Reference (Song, 2014; Van et al., 2015)	Taste description	TAV	
		BT	GT				BT	GT
1	Oxalic acid	5.84 ± 0.84	6.55 ± 0.02	0.31	13	Sour, gentle astringent	19.09	21.41
2	Pyruvic acid	4.17 ± 0.02	4.10 ± 0.01	0.30	13	Sour	13.91	13.68
3	dl‐Tartaric acid	1.90 ± 0.02	1.69 ± 0.01	0.04	13	Sour	43.24	38.52
4	Malonic acid	0.57 ± 0.12	0.58 ± 0	0.07	13	Sour	8.33	8.46
5	Malic acid	2.57 ± 0.04	2.60 ± 0	0.50	14	Sour, gentle astringent	5.14	5.20
6	Lactic acid	10.05 ± 0.05	12.75 ± 0.04	0.30	14	Sour, gentle astringent	33.50	42.49
7	Fumaric acid	0.08 ± 0	0.08 ± 0	0.40	13	Sour, astringent	0.21	0.21
8	Maleic acid	4.65 ± 0	4.68 ± 0	0.35	13	Sour	13.30	13.38
9	Acetic acid	3.95 ± 0.02	3.98 ± 0	0.11	14	Sour, gentle astringent	35.90	36.22
10	Citric acid	8.28 ± 0.06	4.84 ± 0.01	0.13	14	Sour, gentle astringent	63.71	37.23
11	Succinic acid	10.07 ± 0	10.82 ± 0	0.11	14	Sour, light umami	94.96	102.12
12	Gluconic acid	0.37 ± 0	0.48 ± 0	0.02	13	Sour	24.44	32.00

The results showed that the organic acids in BT and GT are mainly oxalic, pyruvic, dl‐tartaric, malonic, malic, lactic, fumaric, maleic, acetic, citric, succinic, and gluconic acids. All of them have a sour taste in common. Additionally, oxalic, malic, lactic, fumaric, acetic, and citric acids are also gently astringent, while succinic acid has a light umami flavor.

Twelve organic acid components were detected in two samples. Among them, the acids with the highest concentrations in BT were succinic (10.07 mg·g^−1^) and lactic (10.05 mg·g^−1^), both of which were greater than 10 mg·g^−1^. The acids in BT with the lowest concentrations were fumaric (0.08 mg·g^−1^), gluconic (0.37 mg·g^−1^), and malonic (0.57 mg·g^−1^), all being lower than 1 mg·g^−1^. The components of the highest concentrations in GT were lactic (12.75 mg·g^−1^) and succinic acids (10.82 mg·g^−1^), and those with the lowest were fumaric (0.08 mg·g^−1^), gluconic (0.48 mg·g^−1^), and malonic acids (0.58 mg·g^−1^). With the exception of equal levels of fumaric acid, the remaining concentrations of acids in GT are greater than those in BT.

Although the organic acid components of the two samples were highly consistent, their concentrations varied greatly. The content and proportion of organic acids may be the main reason for the differences in acidity of the two samples.

### 3.3 Comparison of organic acids’ TAV in BT and GT

According to Table 2, there were 11 organic acids in BT with TAVs greater than 1. This finding indicated that, among the 12 organic acids, only one (fumaric acid) did not contribute to the overall taste. The TAVs of these organic acids, from largest to smallest, were as follows: succinic acid (94.96), citric acid (63.71), dl‐tartaric acid (43.24), acetic acid (35.90), lactic acid (33.50), gluconic acid (24.44), oxalic acid (19.09), pyruvic acid (13.91), maleic acid (13.30), malonic acid (8.33), and malic acid (5.14).

In terms of GT, there were also 11 organic acids with TAVs greater than 1; these organic acids contribute to the overall taste. The TAVs of these organic acids, from largest to smallest, were as follows: succinic acid (102.12), lactic acid (42.49), dl‐tartaric acid (38.52), citric acid (37.23), acetic acid (36.22), gluconic acid (32.00), oxalic acid (21.41), pyruvic acid (13.68), maleic acid (13.38), malonic acid (8.46), and malic acid (5.20).

For taste descriptions, the flavor of succinic acid that contributes the most to the sour taste of BT and GT is sour and light umami; it has the greatest effect on the intensity of the sour taste of the two teas (both teas have a TAV of more than 90). The second highest contribution to the sour taste in BT was citric acid: its taste description was “sour with a gentle astringent.” In GT, the second highest contributor was lactic acid (sour with a gentle astringence). Viewed from another perspective, the content of citric acid in BT is about two times that of GT. Citric acid is the primary reason why BT has a significantly sour taste while GT does not.

The TAV analysis can aid with researching how much organic acids contribute to the overall taste. However, the results of this study showed the similarities among the two different samples, which means the researcher needs to do further tests.

### 3.4 Relationships between sensory and TAV

As far as TAV is concerned, fumaric acid had a TAV of 0.21 for two samples, which means fumaric acid makes no significant contribution to their sour taste. The overall TAV of organic acid in GT was much larger than that in BT, which means the sour taste of GT should be stronger than that of BT; however, in terms of sensory analysis, the sour taste of BT is more remarkable. It is also generally believed that, due to differences in processing technology, BT has a sour taste that GT does not, which contradicts previous research. That means something undetected will play an important role in the overall sensory response. In addition, the undetected role of sour taste in BT and GT will also be studied.

### 3.5 The influence of pH values on the sour taste of different teas

**FIGURE 5:**
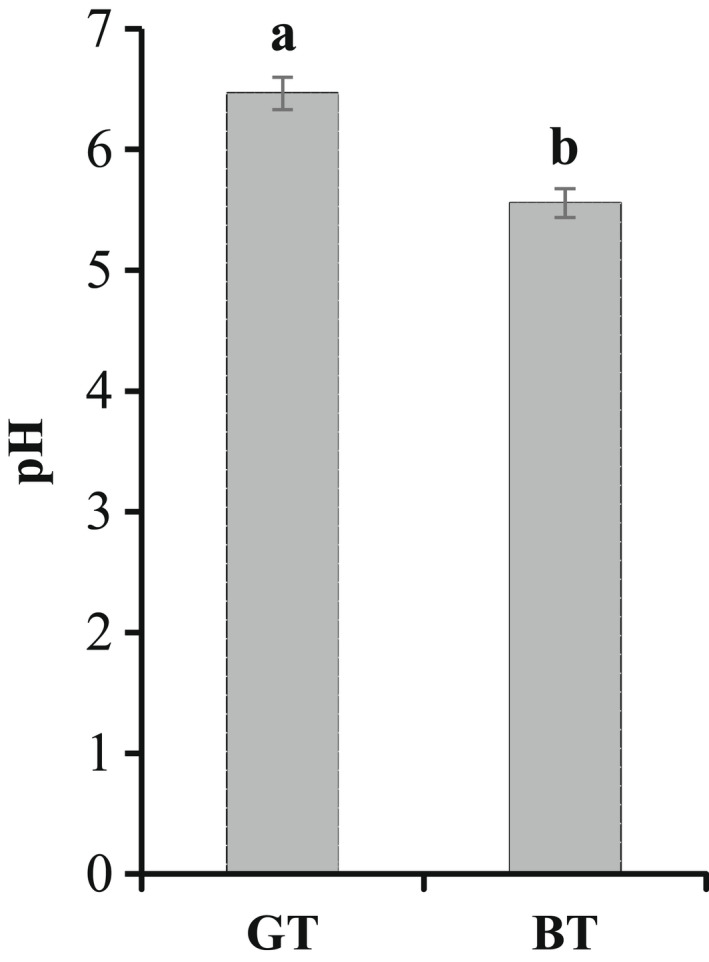
The difference in pH between black tea (BT) and green tea (GT)

The pH values of the two different tea solutions appear in Figure 5. GT has a significantly higher pH than BT; their pH values are 6.47 and 5.58, respectively. According to the sensory analysis results for sour taste obtained in this study, the sour taste of BT is stronger than that of GT. As with studies on the sour taste of beer (Li & Liu, 2015), the lower the pH value, the stronger the sour taste. When the pH value of a GT beverage solution was adjusted to 5.60 with citric acid, the sour taste of GT increased from a pH of 0.33–1.17; therefore, it could be inferred that the pH value is one of the main factors affecting the sour taste of BT and GT. As a result, it would be necessary to adjust pH values before drinking if the sensory sourness of the tea infusions needs to be decreased.

### 3.6 Other factors that may affect the sour taste of tea

Keast and Breslin (2003) studied binary taste–taste interactions, and the researchers determined that, due to the diversity of the amount and structure of chemical substances among the five basic flavors of sweet, sour, bitter, salty, and umami, different combinations of flavor substances in varying concentrations will have different promoting or inhibiting effects. This means that the interaction between the flavoring substances would account for the large difference in sour taste between these two beverages. In general, a high concentration of sweetness, bitterness, and umami and a medium concentration of sweetness suppress the sour taste, whereas a low or medium concentration of bitterness and umami enhances the sour taste. As for the sensory evaluation results of this experiment, a high intensity of bitterness and astringency stands a good chance of suppressing the sour taste of GT. Conversely, a medium concentration of bitterness might well enhance the sour taste of BT.

Stevens and Traverzo (1997) studied the recognition threshold of complex mixtures and found that when more than three flavor substances were mixed together, the taste threshold of the whole solution would be reduced. It is widely known that there are more than one hundred taste substances in tea infusions, which undoubtedly makes it more difficult to study the main substances in each taste type.

## 4 CONCLUSION

In this study, the same ingredients of fresh leaves with identical raw materials and tenderness were processed into GT and BT, respectively. During sensory evaluation, it was found that the acidities of GT and BT were significantly different. Tea flavor comprises aroma and taste, and previous studies have shown that aroma was mainly formed during tea processing, so the difference between GT and BT processing technology is what leads to the variations in the sour taste of the finished teas. The main processing technology of GT is fixation, whereas that of BT is fermentation. The activity of polyphenol oxidase was damaged in the process of making the GT, leading to the inhibition of the enzymatic reaction of tea polyphenols in fresh leaves, which in turn produced more hydrogen ion forms, such as salt, acid, or ester compounds. Although BT, through a series of enzymatic catalysis in the fermentation process, causes hydrogen ions to exist in a free state, the BT tea pH and acidity decrease remarkably. Therefore, the difference in processing may account for the differences in the sour tastes of BT and GT.

### 4.1 Analysis of the causes of sour taste in tea infusion

#### 4.1.1 Interactions between organic acids and other substances

Through this research, it was concluded that the organic acid components are not the only fundamental substances that cause the sour taste in tea infusions. There are two types of organic acids in tea infusions. One contributes to the sour taste of the tea infusion (e.g., lactic, succinic, and citric acids), which are directly involved in the composition of the sour taste. The other type of acid does not contribute to the sour taste, e.g., fumaric acid. These acids play a role in the formation of the sour taste in tea infusions through interactions with other substances. The TAV of the organic acid has little effect on the sour taste of the tea because of the small difference between the types and the content of the organic acids in the two teas.

#### 4.1.2 The pH value

The production of sour taste is due to the dissociation of hydrogen ions (H^+^) by acidic compounds in aqueous solution, which stimulates the taste receptors in the mouth and then transmits information to the taste center of the brain through the neurosensory system (Shao Lixiong & Jianmei, 2020). The concentration of H^+^ directly affects the pH of the tea infusion. The pH of BT is significantly lower than that of GT (the content of free hydrogen ions in BT is more than that of GT). Hence, the intensity of the sour taste is directly related to the pH value of the tea infusion. To follow a pattern within a certain range, the lower the pH value, the stronger the sour taste.

#### 4.1.3 Other sour substances in tea infusion

It is widely believed that gallic acid produced by the hydrolysis of gallated catechins during the withering process and subsequent fermentation might be responsible for the sour taste of BT. It is also believed that the decrease of umami amino acids (such as the anine) and the increase of sour amino acids, such as Asp and Gln, led to an increase in the sour taste of BT. It is the interactions between these complex mixtures that cause such substantial differences in taste between BT and GT. Therefore, it is speculated that the sour taste of tea infusions may be caused by sour amino acids or gallated catechins and not directly related to organic acid components.

### 4.2 The mechanism of the disappearance of the sour taste of tea

A total of 12 kinds of organic acids were detected and screened out in this experiment, and 11 TAVs of organic acids were all more than 1 (1–102.12), which indicated that the above‐mentioned 11 kinds of organic acids contributed to the sour taste of tea, but they were most noticeable in BT. BT had a sourness of 1.33, while GT had almost absent sourness (0.5). The results of the sensory quantitative evaluation of the taste subfactor of tea infusion show that the “sourness” of BT is ubiquitous, while GT has almost no sourness. The possibility of the disappearance of the sour taste was also discussed.

**FIGURE 6:**
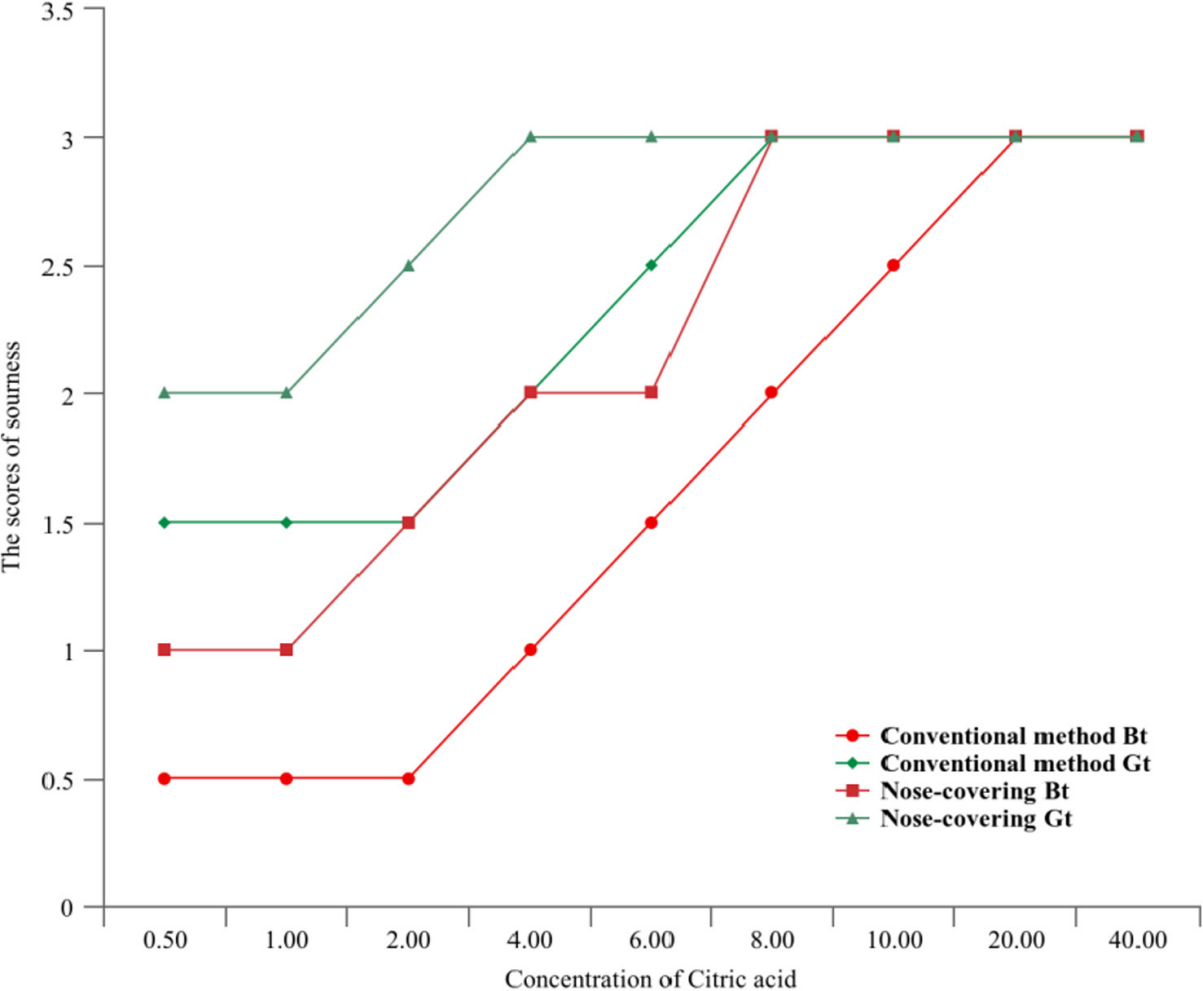
The black tea (BT) and green tea (GT) sour taste change trend chart after adding citric acid. (a) The *X*‐axis represents the concentration of citric acid (mg L^−1^), and the *Y*‐axis represents the acidity score in the tea infusion. (b) The citric acid standard product is directly added to the tea infusion after being prepared according to the proportion. (c) The scores of sourness is consistent with the Table 1. The evaluation was conducted on a 7‐point scale, the number from extreme to absent is 3~0, with each 0.5 divided into a gradient. Sensory evaluation was repeated three times for each evaluator

To verify that the organic acid in tea is not directly related to the sour taste of BT and GT, citric acid standards in different concentrations were added to BT and GT infusion, respectively, and the acidity in the tea infusion was scored by the nose‐covering and conventional methods (see Figure 6). The study found that the sourness in BT gradually increased with the increase of external sour substances, and the sourness increased more obviously with the increase of the concentration of sour substances. After the addition of citric acid, the GT did not immediately show sourness; however, the sourness increased more dramatically with an increase in the concentration of sour substances.

#### 4.2.1 There are other substances in tea soup that inhibit the expression of sour substances

It could be assumed that there are a large number of substances that inhibit the expression of sour taste in tea infusions, and that these substances are higher in GT than BT, thus, BT can have a sour taste while GT does not. As the concentration of externally added sour substances gradually increases, this inhibition effect is reduced, so that the sour taste of BT and GT is gradually revealed after adding external sour substances.

#### 4.2.2 Masking or combined stimuli

Although the concentration of “sour” taste‐stimulating substances in tea infusions is high, the sour taste perception is reduced due to masking or a combined stimulation of other taste substances, and the masking of GT is stronger than that of BT. Existing studies have shown that at lower concentrations, the sour substances gallic acid and ascorbic acid, the bitter substances caffeine, and the umami substances glutamic acid may all affect the sweet (Ruiz et al., 2003). α‐gustducin of the human tongue pathway signaling has an enhancing effect (Boughter et al., 1997; Li, 2006), which may lead to the fact that although the organic acid concentration in tea infusion is higher than the taste threshold, it can still mask the sour taste response under the above effect and enhance other taste effects. Therefore, it is speculated that the disappearance of the sour taste in tea infusions may be due to the masking of other taste substances or the reduction of sour taste perception caused by a combined stimulation.

#### 4.2.3 Effects of retronasal sniffing

There may be aroma substances in the tea infusion that mask the “sour” taste stimulation; however, the evaluators could not perceive the sour because of the complex aroma compounds (e.g., linalool, d‐limonene, β‐cyclocitral) produced by the retronasal sniffing which interfered with the sour taste receptors (see Figure 6). The aroma components can not only result in orthonasal smelling before entering the mouth, but can also be transported to the retronasal passage by airflow from the lungs to produce retronasal sniffing, when swallowing and exhaling (Ruijschop, 2011; Yeretzian et al., 2004). Because the retronasal sniffing stimulation occurs at the same time as chewing, swallowing, and taste perception, it stimulates the prefrontal cortex of the brain and the brain regions related to emotion and memory in a spatiotemporal relationship (Iannilli et al., 2014), which will lead to an important impact on the perception of taste and affect each other (Linscott & Lim, 2016). In addition, studies have shown that the intensity of taste perception differs significantly between using the nose‐covered and conventional methods to taste the same food (Gotow et al., 2018). Therefore, it is speculated that there may be aroma substances in tea infusions that mask the sour taste stimulation, and therefore the evaluators would not detect that the “sour” is the interference of aroma substances from the retronasal sniffing.

## CONFLICT OF INTEREST

The authors declare that they do not have any conflicts of interest.

## ETHICAL APPROVAL

This study was approved by the Local Ethics Committee in China.

## INFORMED CONSENT

Written informed consent was obtained from all study participants.

## Data Availability

All data included in this study are available upon request by contact with the corresponding author.
